# Recurrent solitary fibrous tumor of the pleura with malignant transformation: a case report

**DOI:** 10.1093/jscr/rjab283

**Published:** 2021-07-06

**Authors:** Risa Sakamoto, Tomoka Hamahiro, Ryo Maeda, Takanori Ayabe, Masaki Tomita, Hiroyuki Tanaka

**Affiliations:** Department of Thoracic and Breast Surgery, Faculty of Medicine, University of Miyazaki, Miyazaki, Japan; Department of Thoracic and Breast Surgery, Faculty of Medicine, University of Miyazaki, Miyazaki, Japan; Department of Thoracic and Breast Surgery, Faculty of Medicine, University of Miyazaki, Miyazaki, Japan; Department of Thoracic and Breast Surgery, Faculty of Medicine, University of Miyazaki, Miyazaki, Japan; Department of Thoracic and Breast Surgery, Faculty of Medicine, University of Miyazaki, Miyazaki, Japan; Department of Pathology, Faculty of Medicine, University of Miyazaki, Miyazaki, Japan

## Abstract

We report a rare case of recurrent solitary fibrous tumor (SFT) of the pleura with suspicious malignant transformation. A 78-year-old man had undergone prior surgical resection of the primary and recurrent SFT tumors at 11 and 2 years before the current presentation. Although his primary tumor had a round shape and did not show invasive growth, the current recurrent tumor extended through the neural foramen and had an osteoclastic progression into the thoracic spine. A computed tomography (CT) guided needle biopsy was performed and the pathological diagnosis of the tumor was confirmed as the recurrence of SFT. Immunohistochemically, the MIB-1 proliferation index (Ki-67) of the primary tumor and the current tumor was 1.74 and 30.00%, respectively. These clinical and immunohistochemical findings were strongly suspected the malignant transformation of SFT from benign. He was treated with radiotherapy, and a response was observed.

## INTRODUCTION

Solitary fibrous tumors (SFT) of the pleura originate from the visceral pleura [1–3]. Most of the SFTs are benign tumors, malignant tumors only account for 12% [1–3]. Although most tumors are considered histologically benign, locally aggressive behavior with repeated recurrences and distant metastasis has been observed in some cases [1–3].

Only a limited number of previous literatures reported the histologically confirmed malignant transformation of SFT from benign [4–7]. Here, we report a rare case of recurrent SFT with both clinically and immunohistochemically suspicious malignant transformation. Furthermore, the tumor had a response to external radiotherapy.

## CASE REPORT

**Figure 1:**
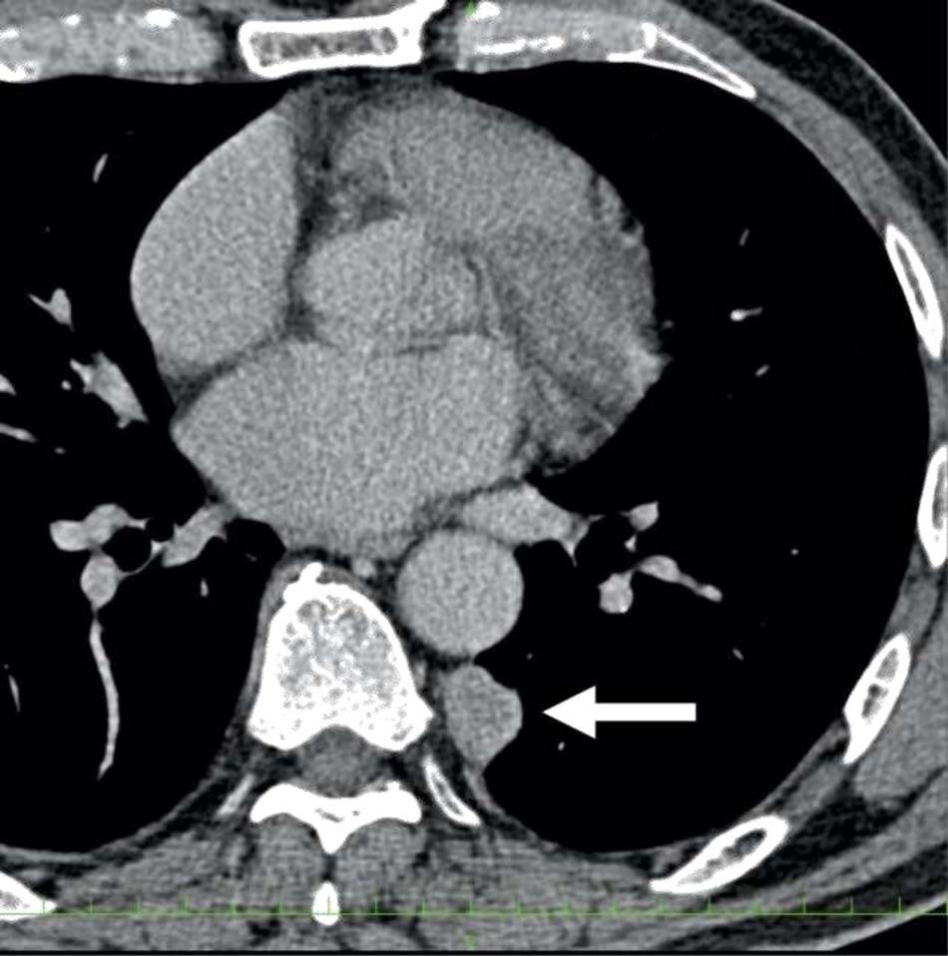
The computed tomographic findings of the primary tumor (arrow).

**Figure 2:**
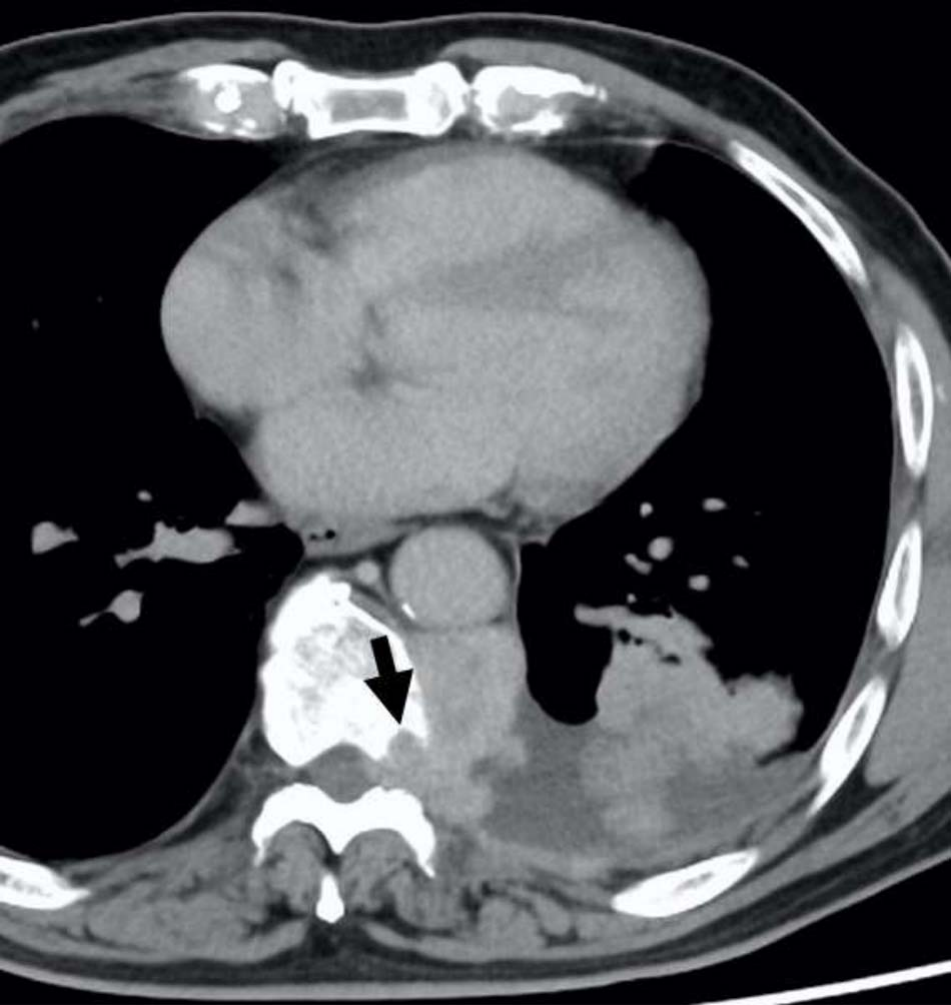
The computed tomographic findings of the current tumor; note the tumor extended through the neural foramen, and had an osteoclastic progression into the thoracic spine (arrow).

**Figure 3:**
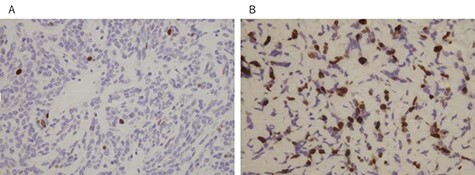
MIB-1 proliferation index (Ki-67) of the primary tumor (**A**) and the current tumor (**B**); (immunostain, original magnification X100).

A 78-year-old man complained of chest pain for 2 months and was admitted to our hospital. Computed tomography (CT) scans revealed a tumor in the left thorax. He had undergone prior surgical resection of the primary SFT and recurrent tumor at 11 and 2 years before the current presentation. The CT findings of the primary tumor revealed a solitary, well-circumscribed and no signs of invasion (Fig. 1). Intraoperatively, the primary tumor arose from the visceral pleura and the resection was performed by wedge resection. The resected tumor was an oval, elastic soft mass with homogenous milky white cut surfaces. Postoperative histopathologic and immunohistochemical findings were consistent with SFT, and the mitosis and malignant features, including nuclear atypia, necrosis or higher cellularity, were hardly seen. In contrast to the primary tumor, as shown in Fig. 2, the CT findings revealed that the current tumor extended through the neural foramen. Furthermore, the tumor had an osteoclastic progression into the thoracic spine. Therefore, this tumor was considered to become malignant, and we considered the surgical complete resection is impossible. For histological confirmation, CT guided needle biopsy was performed. The histological findings of this recurrent tumor were identical to those of the primary tumor. The mitosis was hardly seen in this recurrent tumor. However, immunohistochemically, the MIB-1 proliferation index (Ki-67) of the primary tumor and the current tumor was 1.74 and 30.00%, respectively (Fig. 3A and B).

**Figure 4:**
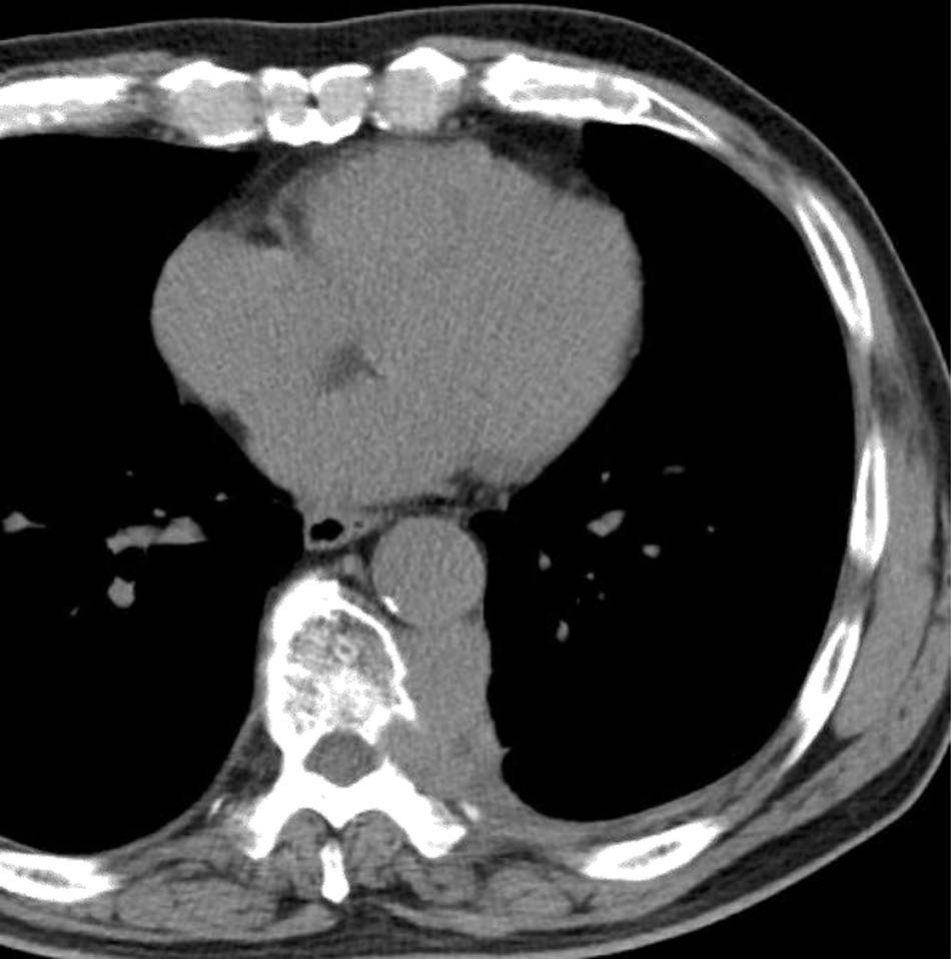
The computed tomographic findings after 6 weeks after radiotherapy.

Since the disease progression might cause paresis, paralysis and loss of bowel/bladder function, palliative thoracic radiotherapy was planned. He received 36 Gray palliative radiotherapy. Six weeks after radiotherapy, the tumor was effectively decreased in size (Fig. 4).

## DISCUSSION

A recurrent SFT of the pleura with malignant transformation is rare. The majority of SFT seems to follow a benign behavior [1–3], but some may recur after surgical resection, and they are considered as malignant transformation [1–3]. The primary tumor of the present case also showed a solitary, well-circumscribed and no signs of an invasion. However, the recurrent tumor of the present case extended through the neural foramen and had an osteoclastic progression into the thoracic spine. These clinical findings suggested the malignant transformation of SFT. Although the recurrence of SFT had been reported previously, only a limited number of previous literatures revealed immunohistochemically confirmed the malignant transformation of SFT from benign [4–7].

Malignant transformation was histologically evaluated the criteria by England *et al.*, included high cellularity, increased mitotic activity (>4 mitotic figures per 10 high power field), pleomorphism, hemorrhage and necrosis [8]. In the present case, these histopathologic features, including mitosis, do not appear to differ between primary and recurrent tumors, despite different radiological findings. However, the MIB-1 proliferation index of the primary tumor and the current tumor was 1.74 and 30.00%, respectively, suggesting the malignant transformation of SFT. MIB-1 is an antibody against Ki-67 which is a protein associated with cell proliferation and ribosomal RNA synthesis, thereby MIB-1 is a reliable immunohistochemical marker of cell proliferation [9]. Sun *et al.* reported that Ki-67 is diagnostically relevant to the evaluation of malignant SFT and this protein is thought to be a potentially useful marker for the prognosis of SFT [9].

The mechanism of the development of malignant SFT has not been fully elucidated. Yokoi *et al.* suggested that malignant SFTs develop mainly in two ways; one is malignant or high-grade transformation within a benign, low-grade or intermediate-grade SFT and the other is de novo occurrence of malignant SFT [7]. Further studies of the mechanism of development of malignant transformation are warranted.

Surgical exploration allows the establishment of a cure for SFT. However, the role of the other treatment modalities, such as radiotherapy and chemotherapy, in the management is unclear because of the rarity of the disease and the successful results of the surgical treatment. Since the present case had low mitotic activity, the response to radiotherapy of the tumor was skeptical. However, the present tumor had a response to radiotherapy. This clinical course might also indicate the malignant transformation and aggressiveness of the tumor. Saynak *et al.* also demonstrated the significant response of the pleural SFT to radiotherapy and reported that radiotherapy for SFT may be considered and be helpful if the surgery is impossible or refused by the patients [10].

## CONCLUSION

We report a case of immunohistochemically confirmed malignant transformation of recurrent pleural SFT. Furthermore, this recurrent tumor had a response to radiotherapy.

## CONFLICT OF INTEREST STATEMENT

None declared.
